# The complete mitochondrial genome of *Papilio xuthus* (Lepidoptera: Papilionidae) and implications for Papilioninae taxonomy

**DOI:** 10.1080/23802359.2016.1144101

**Published:** 2016-03-28

**Authors:** Yunhe Wu, Jie Song, Naiyi Liu, Zhengqing Cai, An Yulin, Jie Fang

**Affiliations:** a School of Life Sciences, Anhui University, Hefei, Anhui, PR China;; b Nanjing Entry-Exit Inspection and Quarantine Bureau of China, Nanjing, PR China;; c College of Forest, Nanjing Forestry University, Nanjing, PR China;; d Plant Quarantine Laboratory of Jiangsu Entry-Exit Inspection and Quarantine Bureau of China, Nanjing, PR China

**Keywords:** Mitochondrial genome, *Papilio xuthus*, phylogenetic analysis

## Abstract

The complete mitochondrial genome of *Papilio xuthus* (Lepidoptera: Papilionidae) was determined in this study. The mitochondrial genome is a circular molecule of 15 359 and contains 37 genes including 13 protein-coding genes (PCGs), 22 transfer RNA genes, two ribosomal RNA genes and one control region. The nucleotide composition of the *A. chinensis* mitogenome is strongly biased toward A + T nucleotides (80.45%). Nine protein-coding genes and 14 tRNA genes are encoded on the H strand, and the other four protein-coding genes and eight tRNA genes are encoded on the L strand. The gene order and the orientation of their mitogenomes were similar to all know Papilionidae species. Finally, the phylogenetic relationships of 11 Papilionidae species were reconstructed based on complete mitochondrial genome using the Bayesian inference (BI) and the maximum-likelihood (ML) method. These molecular-based phylogenies support the traditional morphologically based view of relationships within the Papilionidae.

The papilionid butterflies, including about 530 species, are distributed worldwide (Yagi et al. 1999). As many of them have large beautiful wings, have greatly contributed to studies of ecology, behavior and evolution in insects (Scriber 1995; Boggs et al. 2003). The butterfly *Popilio xuthus* mainly distributed in East Asia (Li et al. 2015) and the larva is a famous pest of agriculture (Kong et al. 2006). The specimen was collected in the Shengjin Lake National Nature Reserve, Anhui province, China, in August 2015. Total genomic DNA was extracted from muscle tissue using the standard phenol–chloroform protocol (Sambrook et al. 2001). The mitochondrial genome was amplified by polymerase chain reaction (PCR) using five pairs of primers. Except for the A + T-rich region, the PCR fragments were directly sequenced by primer walking. The A + T-rich region of lepidopterans contains several features that obscure proper base calling, so the PCR production was sequenced after cloning.

The mitochondrial genome is a circular molecule of 15 359 bp (accession no. KU356933) and contains 37 genes including 13 protein-coding genes (PCGs), 22 transfer RNA genes, two ribosomal RNA genes and one control region. Among these, 14 genes were encoded on the L-strand, including four PCGs (*ND1*, *ND4*, *ND4L* and *ND5*), two rRNA genes, eight tRNA genes (*tRNA^Glu^*, *tRNA^Cys^*, *tRNA^Tyr^*, *tRNA^Phe^*, *tRNA^His^*, *tRNA^Pro^*, *tRNA^leu(CUN)^* and *tRNA^Val^*) and A + T-rich region. The remaining 23 genes are encoded on the H strand. The arrangement of genes is similar to all know Papilionidae species.

Eleven Papilionidae species were used to reconstruct phylogenetic tree based on the complete mitochondrial genome through the Bayesian inference (BI) and maximum-likelihood (ML) methods, using *Timelaea maculate* and *Argyres hyperbius* as the outgroup. MrBayes Version 3.1.2 (Ronquist & Huelsenbeck 2003) and a PHYML online web server (Guindon & Gascuel 2003; Guindon et al. 2005) were employed to reconstruct BI and ML trees, respectively. For BI analysis, the model GTR + I+G was selected via MrModeltest version 2.1 (Nylander et al. 2004), the MCMC was run for 1 000 000 generations until the average standard deviation of split frequencies reached a value less than 0.01, with the Bayesian posterior probabilities calculated from the sample points after the MCMC algorithm had started to converge (Zhan & Fu 2011). The result of phylogenetic tree among the 11 Papilionidae species is shown in Figure 1. It is clearly seen that the phylogenetic tree is divided into two major clades. The first lineage, subfamily Papilioninae, includes species of genus *Papilio* (*Papilio bianor*, *Papilio maackii*, *Papilio machaon*, *P. xuthus*, *Papilio polytes* and *Papilio helenus*), *Graphium* (*Graphium chironides* and *Graphium timur*), *Troides* (*Troides aeacus*). The subfamily Zerynthiinae (*Sericinus montela* and *Luehdorfia taibai*) forms the second clade and is sister to subfamily Papilioninae. These molecular-based phylogenies support the traditional morphologically based view of relationships within the Papilionidae (Wu et al. 2001).

**Figure 1.**
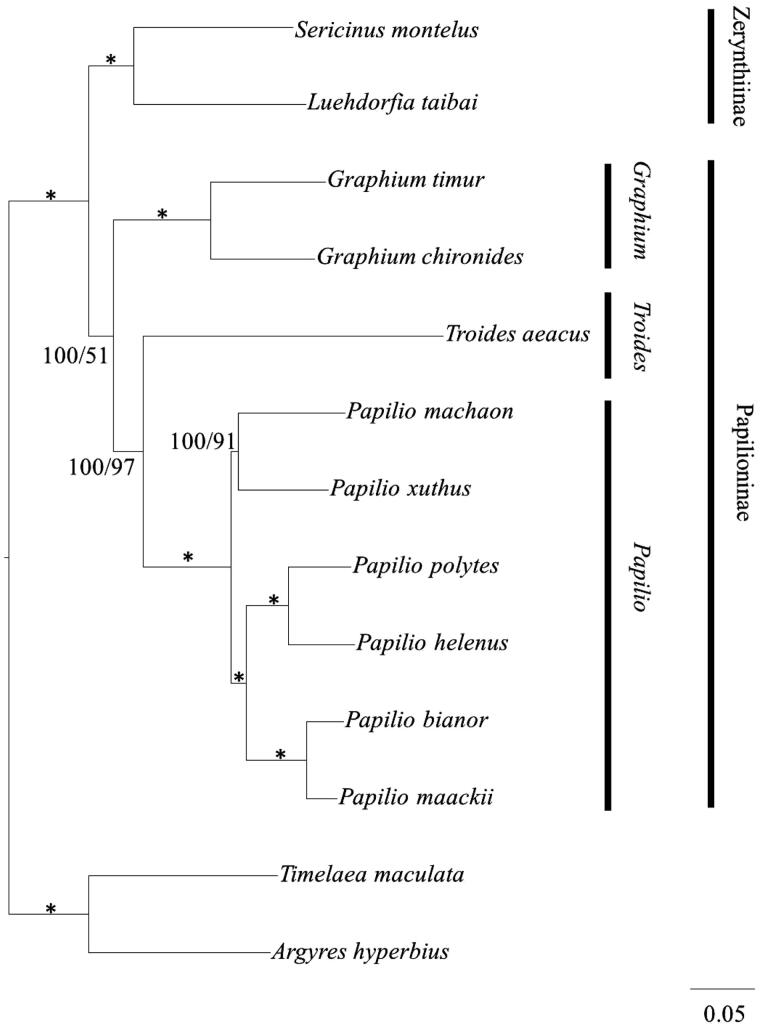
Inferred phylogenetic relationships among Papilionidae based on the complete mitochondrial genome using Bayesian inference (BI) and maximum-likelihood (ML) analysis. Branch lengths and topologies came from the Bayesian analyses. Numbers in each branch indicate Bayesian posterior probabilities (BPP)/maximum-likelihood (ML) bootstrap values. *Indicates posterior probabilities = 100 and ML bootstrap = 100. GenBank accession numbers for the published sequences are NC018040.1 (*Papilio bianor*), NC021411.1 (*Papilio maackii*), NC018047.1 (*Papilio machaon*), KU356933 (*Papilio xuthus*), NC024742.1 (*Papilio polytes*), NC026910.1 (*Graphium chironides*), NC024098.1 (*Graphium timur*), EU625344.1 (*Troides aeacus*), HQ259122.1 (*Sericinus montela*), NC023938.1 (*Luehdorfia taibai*), NC025757.1 (*Papilio helenus*), NC021090.1 (*Timelaea maculate*), NC015988.1 (*Argynnis hyperbius*).

Our study of *P. xuthus* can provide a useful database for analyzing the classification and status in Papilionidae. In addition, it is useful to construct molecular identification of this species.
